# Surface and buildup dose characteristics for 6, 10, and 18 MV photons from an Elekta Precise linear accelerator

**DOI:** 10.1120/jacmp.v4i1.2537

**Published:** 2003-01-01

**Authors:** Eric E. Klein, Jacqueline Esthappan, Zuofeng Li

**Affiliations:** ^1^ Division of Physics Department of Radiation Oncology Washington University School of Medicine Campus Box 8224, 660 S. Euclid Ave. St. Louis Missouri 63110‐1093

**Keywords:** buildup region, surface dose, Elekta, radiotherapy

## Abstract

Understanding head scatter characteristics of photon beams is vital to properly commission treatment planning (TP) algorithms. Simultaneously, having definitive surface and buildup region dosimetry is important to optimize bolus. The Elekta Precise linacs have unique beam flattening filter configurations for each photon beam (6, 10, and 18 MV) in terms of material and location. We performed a comprehensive set of surface and buildup dose measurements with a thin window parallel‐plate (PP) chamber to examine effects of field size (FS), source‐to‐skin distance (SSD), and attenuating media. Relative ionization data were converted to fractional depth dose (FDD) after correcting for bias effects and using the Gerbi method to account for chamber characteristics. Data were compared with a similar vintage Varian linac. At short SSDs the surface and buildup dose characteristics were similar to published data for Varian and Elekta accelerators. The FDD at surface $\left({\text{FDD}}_{0}\right)$ for 6, 10, and 18 MV photons was 0.171, 0.159, and 0.199, respectively, for a $15\times 15{\;\text{cm}}^{2}$, 100 cm SSD field. A blocking tray increased ${\text{FDD}}_{0}$ to 0.200, 0.200, and 0.256, while the universal wedge decreased ${\text{FDD}}_{0}$ to 0.107, 0.124, and 0.176. ${\text{FDD}}_{0}$ increased linearly with FS (~1.16%/cm). ${\text{FDD}}_{0}$ decreased exponentially for 10 and 18 MV with increasing SSD. However, the 6 MV ${\text{FDD}}_{0}$ actually increased slightly with increasing SSD. This is likely due to the unique distal flattening filter for 6 MV The measured buildup curves have been used to optimize TP calculations and guide bolus decisions. Overall the ${\text{FDD}}_{0}$ and buildup doses were very similar to published data. Of interest were the relatively low 10 MV surface doses, and the 6 MV ${\text{FDD}}_{0}$'s dependence on SSD. © 2003 *American College of Medical Physics.*

PACS number(s): 87.53.–j, 87.66.–a

## INTRODUCTION

Understanding head scatter characteristics of photon beams is important to properly commission and test treatment planning (TP) algorithms. Most modern commercial TP systems (TPS) either use model‐based or analytical methods to calculate dose distributions in the buildup region. In either case it is vital to measure buildup curves accurately in order to evaluate the accuracy of TPS calculations. Simultaneously, having definitive dosimetry at the surface and buildup region is important to optimize bolus thickness required to enhance surface dose, in clinical cases such as inflammatory breast disease. Finally, it is imperative to describe the effect of scattering (wedge, collimating jaws, etc.) and immobilization (polyurethane foam, treatment tables, etc.) media on surface dose.

Comprehensive data sets in the buildup and build‐down (exit) regions have been published for vintage Varian^1^–^4^ and Siemens^5^ linacs, and for modern day Varian^6^–^9^ or Elekta^10^ linacs. In these publications, it is evident that subtle differences in the unique beam delivery systems can affect dose to the buildup region. These include the beam monitor chamber and flattening filter construction.

**Figure 1:**
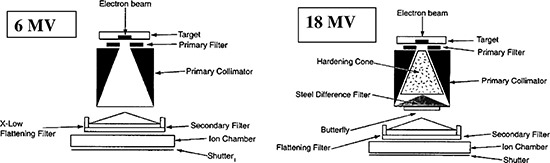
Photon delivery system for Elekta Precise linacs. (a) 6 MV system that is unique by virtue of the x‐low flattening filter relatively distal from the primary filter. (b) Expanded view of the 18 MV system unique by virtue of the relatively proximal steel difference filter.

In addition, support devices such as treatment tables or polyurethene immobilization platforms can also influence surface dose.^2^,^8^,^9^,^11^,^12^ The Elekta Precise linear acccelerators (Elekta, Norcross, GA) have unique beam flattening filter configurations for each photon beam (6, 10, and 18 MV) in terms of material and location. The filter configurations for the 6 and 18 MV photon beams are shown in Fig. 1.

We performed a comprehensive set of surface and buildup dose measurements on the Precise linacs with a thin window parallel‐plate chamber to examine effects of field size (FS), source‐to‐skin distance (SSD), and attenuating media.

## METHODS AND MATERIALS

The Elekta Precise linacs in our clinic deliver 6, 10, and 18 MV photons with ${\text{PDD}}_{10}$ of 68.3%, 73.1%, and 79.5%, respectively. Measurements were performed with a parallel plate (PP) ionization chamber (PS‐033, Capintec, Ramsey, NJ) possessing an entry window thickness of $0{.5\;\text{mg}/\text{cm}}^{2}$, a plate separation of 2 mm, and a collecting diameter of 16.2 mm. For each measurement point, the relative ionization was acquired by dividing the charge collected at depth, via a modified Keithley electrometer (Modified K602, CNMC Co., Nashville, TN), by the charge at the depth of $d_{\text{max}}$ and then corrected to PDD by correcting for bias effects and using the Gerbi method to account for chamber characteristics.^13^ This was accomplished in the following method: *Bias Correction*: All ionization readings were corrected by first accounting for bias effects where,

(i)

(1) $$\left|\frac{M^{+}+M^{-}}{2}\right|=M,$$

where $M^{+}$ and $M^{-}$ are the collected positive and negative charges, respectively. The uncorrected percent depth ionization (PDI) is calculated from the uncorrected bias averaged ionization readings.

(ii)

(2) $$PDI=\frac{M(d)}{M(d_{max})}.$$

The PDI was then corrected to PDD by accounting for chamber (Capintec Parallel‐Plate) characteristics according to the Gerbi method.

(iii)

(3) $$PDD=PDI-\xi (0,E)le^{-\alpha (d/d_{max})},$$

where ξ(0,E)=energy dependent chamber corrections,

ξ(0,E)=[−1.666+(1.982IR)]×[C−15.8),

where

$$\begin{array}{c} \text{IR}=\text{ionization ratio}\;(6\;\text{MV}=0.675,\;10\;\text{MV}=0.728,\;18\;\text{MV}=0.783)\\ C=\text{sidewall}-\text{collector}\;\text{distance}\;(\text{mm})=6\\ \alpha =\text{constant of}\;5.5. \end{array}$$

Buildup region data were measured for field sizes ranging from 5 to 40 cm, SSDs ranging from 80 to 140 cm, and for depths from surface to just beyond $d_{\text{max}}$. Field sizes were defined by the collimator setting (100 cm SSD). In addition, surface doses were measured with the Elekta *Universal* wedge and a 9 mm lexan block tray in place. Data were compared with a similar vintage Varian linac.

## RESULTS

### A. Surface dose characteristics

At short SSDs (⩽100cm) the surface and buildup dose characteristics were similar to previously published data. The data are reported in fractional depth dose data (PDD/100), as this was the reporting method in prior publications. The fractional depth dose (FDD) at surface $\left({\text{FDD}}_{0}\right)$ for open 6, 10, and 18 MV photon beams was 0.171, 0.159, and 0.199, respectively, for a $15\times 15{\;\text{cm}}^{2}$, 100 cm SSD field. The blocking tray increased ${\text{FDD}}_{0}$ to 0.200, 0.200, and 0.256, while the universal wedge decreased ${\text{FDD}}_{0}$ to 0.107, 0.124, and 0.176 for the 6, 10, and 18 MV photons, respectively. Table I summarizes this data along with a comparison to Varian data. The wedge data in each case were measured without the respective block trays in place. The Elekta wedge is the *Universal* wedge with its proximal surface located at 18.6 cm from the source; and the Elekta block tray is a 9 mm lexan tray, located at 64.7 cm from the source. The Varian wedge is a 45° lead wedge (located at 49.2 cm from the source); and the Varian block tray is a 6 mm lexan tray, located at 61.6 cm from the source.

**Table I:** Elekta precise fractional surface dose: $15\times 15{\;\text{cm}}^{2}$ field size, 100 cm SSD. Values in parentheses are for a Varian 2100C accelerator.

Absorber/Energy	6 MV	10 MV	18 MV
Open	0.171 (.205)	0.159	0.199 (0.215)
Block Tray	0.200 (.226)	0.200	0.256 (0.223)
Universal Wedge (Varian 45° wedge)	0.107 (.179)	0.124	0.176 (0.136)

### B. Dependence on field size

**Figure 2:**
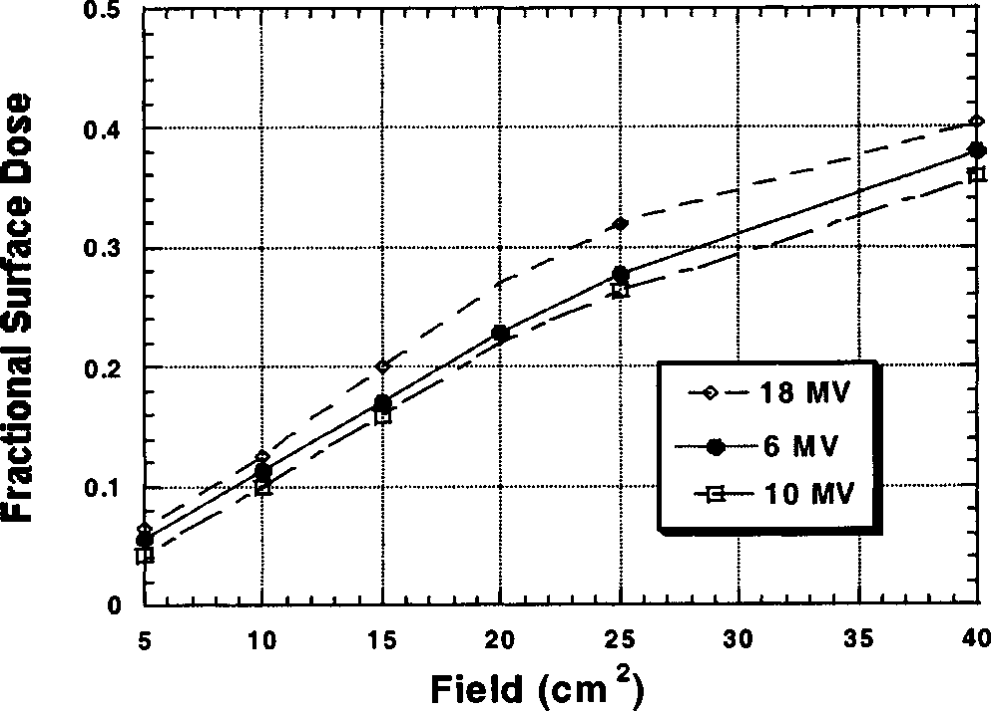
Fractional surface dose as a function of energy and field size at a constant SSD of 100 cm, without a block tray in place.

We found that the surface dose, reported as FDD, increased nearly linearly with FS (~1.16%/cm). The graph in Fig. 2 depicts this trend.

### C. Dependence on SSD

**Figure 3:**
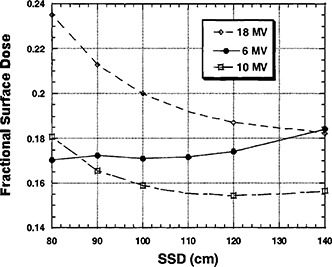
Fractional surface dose as a function of energy and SSD with a constant collimating field size of 15×15 cm, and without a block tray in place.

The ${\text{FDD}}_{0}$ decreased exponentially for 10 and 18 MV with increasing SSD. However, the 6 MV ${\text{FDD}}_{0}$ actually increased slightly with increasing SSD. This is likely due to the unique distal “x‐ray” flattening filter for 6 MV. The graph in Fig. 3 summarizes these results.

### D. Buildup data

**Figure 4:**
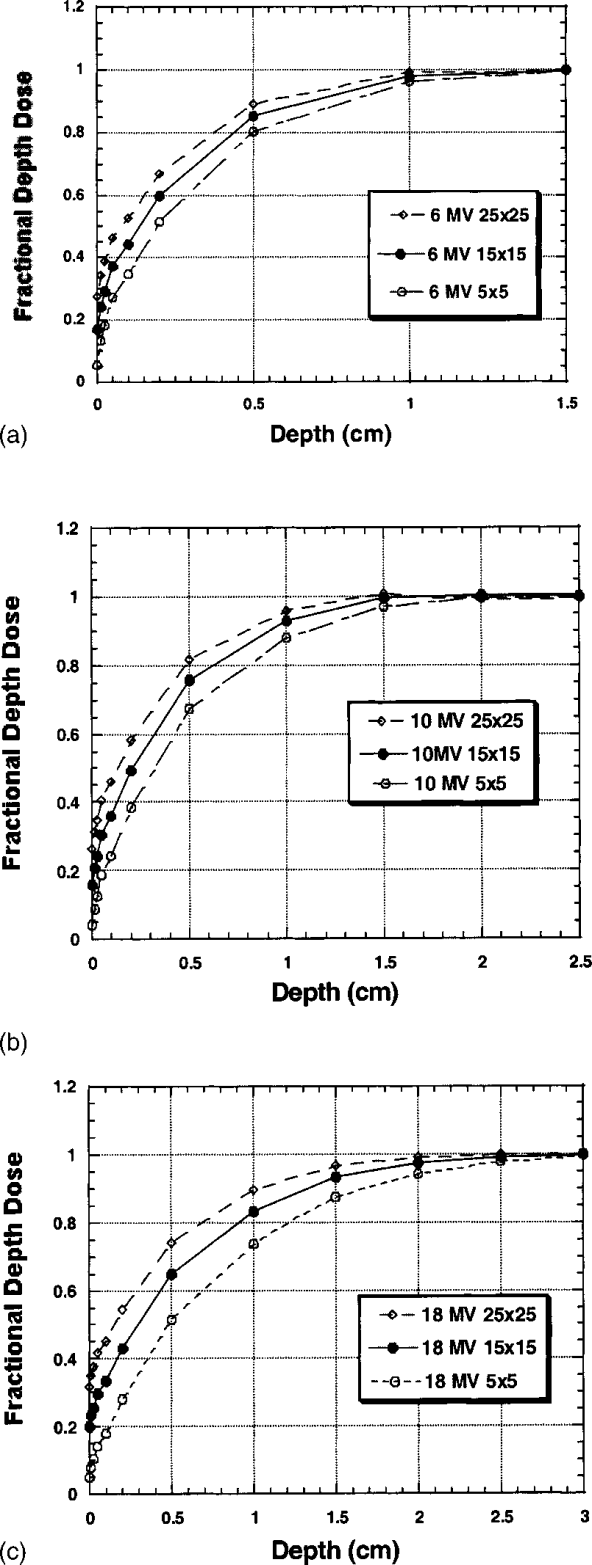
(a) Fractional depth dose as a function of depth and field size at a constant for 6 MV photons, without a block tray in place. (b) Fractional depth dose as a function of depth and field size at a constant for 10 MV photons, without a block tray in place. (c) Fractional depth dose as a function of depth and field size at a constant for 18 MV photons, without a block tray in place.

Figures 4 graphically represent the buildup curves for each of the photon energies. The buildup curves are normalized to the respective $d_{\text{max}}$ FDD values.

**Figure 5:**
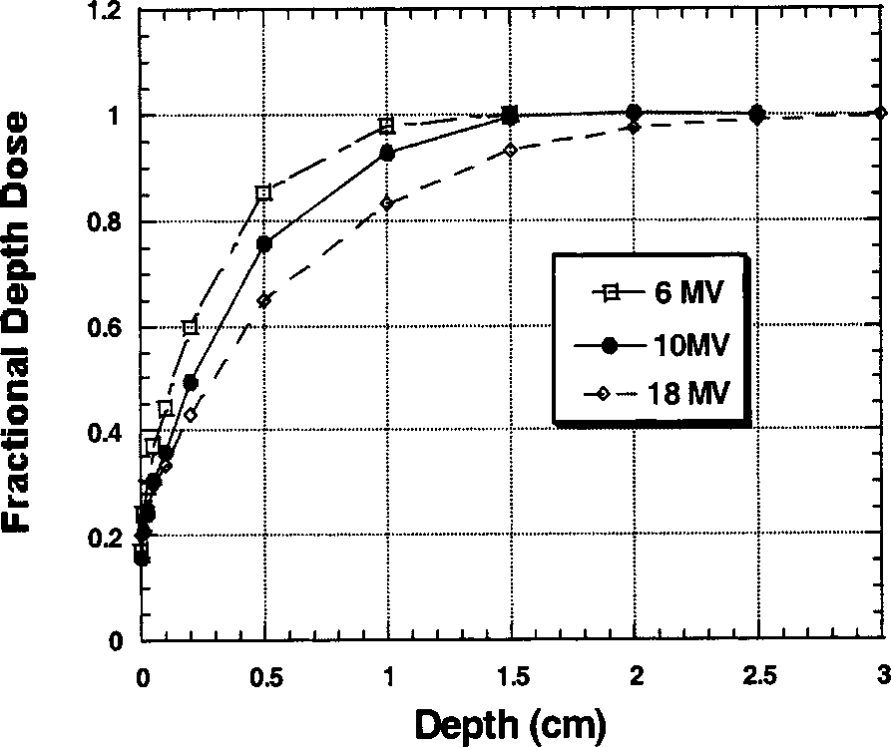
Fractional depth dose as a function of depth and energy for a 15×15 cm field size, without a block tray in place.

The $15\times 15{\;\text{cm}}^{2}$ data for each energy were extracted for comparison. Once again data are normalized to each respective $d_{\text{max}}$ value. The results are displayed in Fig. 5.

## DISCUSSION AND CONCLUSIONS

The measured buildup curves have been used to validate TP calculations and guide bolus decisions. Overall the FDD at surface $\left({\text{FDD}}_{0}\right)$ and buildup doses were very similar to published data. One point of interest is the relatively low 10 MV surface doses. This is most likely due to the two‐tier flattening filter system employed for the 10 MV beam. Also of special interest was the 6 MV dependence of ${\text{FDD}}_{0}$ on SSD. The constant ${\text{FDD}}_{0}$, independent of SSD, is likely a reflection of the distal location of the 6 MV flattening filter, placing the source of scattered electrons and photons further from the target and thereby creating a smaller solid angle of head scatter. In terms of dependence on attenuation devices, it is of interest that the influence of the block tray on surface was minimal for 6 MV beams compared with prior reports, with only a minimal increase (3% absolute increase), but was consistent compared with prior reports for 18 MV photons (6% absolute increase). This is due to the relatively thick (9 mm) lexan tray compared with other machines' 6 mm trays. This thick tray absorbs a greater percentage of low energy scattered electrons and photons for 6 MV photons compared to the 18 MV beam. This same scenario is even more evident for the steel universal wedge whereby the wedge acts as both an generator and absorber of head scatter. The result is a greater absorption for 6 MV compared with 18 MV photons. In general, the Precise linac delivers lower surface dose for 6 and 18 MV photons compared with a similar vintage Varian machine, with the exception of attenuated 18 MV photons.
